# Real-time, non-destructive and in-field foliage yield and growth rate measurement in perennial ryegrass (*Lolium perenne* L.)

**DOI:** 10.1186/s13007-019-0456-2

**Published:** 2019-07-10

**Authors:** Kioumars Ghamkhar, Kenji Irie, Michael Hagedorn, Jeffrey Hsiao, Jaco Fourie, Steve Gebbie, Valerio Hoyos-Villegas, Richard George, Alan Stewart, Courtney Inch, Armin Werner, Brent Barrett

**Affiliations:** 1 Forage Science, Grasslands Research Centre, AgResearch, Palmerston North, New Zealand; 2 Lincoln Agritech Ltd, Lincoln, New Zealand; 3 Red Fern Solutions Ltd, Christchurch, New Zealand; 4 Development Engineering, Lincoln Research Centre, AgResearch, Lincoln, New Zealand; 5 Forage Science, Lincoln Research Centre, AgResearch, Lincoln, New Zealand; 6 PGG Wrightson Seeds Ltd, Hamilton, New Zealand; 7 PGG Wrightson Seeds Ltd, Christchurch, New Zealand; 8 New Zealand Agriseeds Ltd, Christchurch, New Zealand

**Keywords:** Biomass, Dry matter, Pasture, Yield, Grass, Growth rate, LiDAR, *Lolium perenne*, Perennial ryegrass

## Abstract

**Background:**

In-field measurement of yield and growth rate in pasture species is imprecise and costly, limiting scientific and commercial application. Our study proposed a LiDAR-based mobile platform for non-invasive vegetative biomass and growth rate estimation in perennial ryegrass (*Lolium perenne* L.). This included design and build of the platform, development of an algorithm for volumetric estimation, and field validation of the system. The LiDAR-based volumetric estimates were compared against fresh weight and dry weight data across different ages of plants, seasons, stages of regrowth, sites, and row configurations.

**Results:**

The project had three phases, the last one comprising four experiments. Phase 1: a LiDAR-based, field-ready prototype mobile platform for perennial ryegrassrecognition in single row plots was developed. Phase 2: real-time volumetric data capture, modelling and analysis software were developed and integrated and the resultant algorithm was validated in the field. Phase 3. LiDAR Volume data were collected via the LiDAR platform and field-validated in four experiments. Expt.1: single-row plots of cultivars and experimental diploid breeding populations were scanned in the southern hemisphere spring for biomass estimation. Significant (*P *< 0.001) correlations were observed between LiDAR Volume and both fresh and dry weight data from 360 individual plots (R^2^ = 0.89 and 0.86 respectively). Expt 2: recurrent scanning of single row plots over long time intervals of a few weeks was conducted, and growth was estimated over an 83 day period. Expt 3: recurrent scanning of single-row plots over nine short time intervals of 2 to 5 days was conducted, and growth rate was observed over a 26 day period. Expt 4: recurrent scanning of paired-row plots over an annual cycle of repeated growth and defoliation was conducted, showing an overall mean correlation of LiDAR Volume and fresh weight of R^2^ = 0.79 for 1008 observations made across seven different harvests between March and December 2018.

**Conclusions:**

Here we report development and validation of LiDAR-based volumetric estimation as an efficient and effective tool for measuring fresh weight, dry weight and growth rate in single and paired-row plots of perennial ryegrass for the first time, with a consistently high level of accuracy. This development offers precise, non-destructive and cost-effective estimation of these economic traits in the field for ryegrass and potentially other pasture grasses in the future, based on the platform and algorithm developed for ryegrass.

**Electronic supplementary material:**

The online version of this article (10.1186/s13007-019-0456-2) contains supplementary material, which is available to authorized users.

## Background

It is important to accurately measure vegetative mass in crop and pasture plants, given the significance of this characteristic in global agricultural productivity and food supply [1, 2]. Rapid collection of accurate information on large numbers of experimental units is needed to inform plant breeding and adaptation. Precise measurement of vegetative mass, and more specifically fresh weight (FW) and dry weight (DW), will improve understanding of foliage yield and more complex traits such as drought tolerance [3], water use efficiency [4] and salinity tolerance [5] and assists in detecting association between molecular markers and these traits [6].

Vegetative mass is associated with important traits such as grain yield in cereals, and herbage dry matter yield (DMY, kg/ha) accumulation over time in perennial pasture grasses. Grasslands cover 40% of earth’s temperate and tropical terrestrial surface, an estimated 52.5 M km^2^ [7]. In managed landscapes, improved varieties of perennial grass provide amenity, anchor and enhance soil, and nutrition for ruminant animal production.

The dietary, environmental and economic value of forage in livestock farming performance and profitability have been reviewed by Wangchuk et al. [8], Long and Ketterings [1] and Boone et al. [9]. Reliable and rapid pasture plant growth is essential for optimal performance of livestock because it provides dry matter nutrition to the animal for conversion to milk, meat and fibre [10, 11].

For perennial ryegrass (PRG), estimated DMY gains of 4% to 5% per decade in the recent decades [12] and 0.75% per annum since 1990 [13] have been achieved. These lag behind the rate of gain in livestock and crops such as wheat and maize, where breeding for harvest index and plant density have led to substantial yield gains [14, 15]. To accelerate the rate of genetic gain for DMY in perennial grasses, there is a need to develop more efficient tools for breeding, including rapid, reliable non-invasive means to measure vegetative growth over time [16].

In pasture grasses, vegetative DMY is an inherently complex trait, making it challenging to accurately measure and apply selection pressure in breeding programmes [17]. Furthermore, the cyclical nature and morphological diversity and plasticity of dry matter accumulation over time creates substantial complexity for scientists, bioinformaticians, and modelling experts, requiring any phenomics model to be able to optimise vegetative mass measurement for precision and accuracy to better estimate DMY under a wide range of conditions.

At the individual plant level, DMY is the sum of edible leaf and pseudostem morphometric mass per unit area, and is influenced by tiller density, leaf shape, dry matter content, and leaf elongation rate as well as underpinning physiological factors such as light interception, water use efficiency and tolerance to biotic and abiotic stresses [18, 19]. As plants age, they may expand or contract their vegetative parts as a function of rates of tiller formation and death [20–22].

While it is possible to measure DMY in individual plants, there is a low correlation between individual plant FW or DW and sward DMY in perennial grasses [23, 24]. Given that grass used for amenity and economic purposes is generally grown in solid swards of one or more herbaceous species, the most informative measures of DMY in forage grasses rely on environments with high levels of competition among plants, with plants grown in rows or small swards for evaluation by plant breeders and agronomists. The gross morphological similarity coupled with extreme proximity and intermingling of individual plants makes it impossible to collect data from individual plants in a row or sward [25, 26]. Thus plant breeders often rely on the use of maternal families or bulked populations for evaluation and selection, giving rise to breeding strategies where large scale field trials of single row or small sward plots are used to monitor patterns of FW or DW accumulation.

Standard methods to estimate DMY in plots of forage grasses involve destructive mechanical harvest followed by drying and weighing to record DW; or an indirect estimate based on a non-destructive visual preference score assessed by an experienced plant breeder. However, reliance on visual preference or destructive harvests limits breeder’s ability to cost effectively and precisely assess DMY in large scale or multi-site trials. Furthermore, the ability to measure growth rate, especially at high temporal resolution, is seriously constrained by current technology and is not used in pasture plant breeding, despite the obvious economic implications of the trait. The use of destructive mechanical harvests also precludes grazing, which is important when selecting or evaluating pasture plants [27, 28].

For these reasons, accurate, non-destructive and efficient estimation of accumulated DMY and growth rate in single rows and small plots are substantial bottlenecks that hamper plant breeder’s efforts to enhance the rate of gain for DMY [29], despite it being the highest priority trait for the pastoral sector [10].

Phenomics, use of sensors and computing techniques, enable complete phenotyping of traits and response to genetic and environmental influence over time. Mobile, field-based phenomics platforms using sensors and computers may assist breeders, agronomists and other researchers to implement this high-throughput trait phenotyping in scenarios relevant for evaluating, selecting and managing improved populations. Existing imaging and sensor technologies for estimating vegetative mass use a broad range of electro-optic wavelengths [30–33]. Due to its low-cost as well as similarity with color detection range by humans (~ 400–700 nm), visual imaging or Red–Green–Blue (RGB) imaging using commercial or industrial smart cameras is used for analyzing plant forms, from individual leaf phenotyping [34] to shoot architecture [35] and yield measurement [36]. However, RGB imaging for yield measurement has its downsides such as its dependence on external light source, and the need to at least one camera to go beyond surface measurement and be able to estimate depth and therefore the complexity of the integration of data between at least two cameras [36]. This will limit the high quality RGB-based yield measurement to contained environments if used alone and without other sensors. Hyperspectral imaging for forage yield estimation in legume and grass swards has been reported by [37]. Whilst correlations have been observed between spectral measurements converted to indices and vegetative biomass [38], they tend to only reflect partial information, partly influenced by reasons such as capturing information solely from surface reflectance data. Most studies involving spectroscopic measurement of biomass use various vegetation indices inferred from properties of chlorophyll, but these indices become uninformative as biomass increases [39]. Jin et al. [40], however, showed in a cereal crop that the “normalized difference matter index” to be the spectral index with highest correlation with biomass (R^2^ = 0.77).

Light detection and ranging (LiDAR) is a well-established sensor technology, and promising tool for estimation of plant biophysical traits [41, 42] and detailed physical characterization of plants in the field [43, 44]. It is also capable of precisely mapping below canopy soil [45, 46]. It has been shown to contribute to multi-sensor systems for evaluation of pasture grasses [47, 48] but has not been tested as a stand-alone system in forage vegetative DMY assessment. LiDAR is a dynamic sensing system that both emits and captures frequent impulsive laser signals. Unlike traditional cameras, LiDAR scanners directly acquire distance and distribution data [49].

Ground-based LiDAR has been shown to be a good estimator of crop density in small grain crops (with R^2^ of 0.80–0.96) [50], biomass in grapevine (average Pearson’s product moment correlation coefficient of 0.93) [51], crop dry weight (RMSE ≤ 0.68) [52] and biomass in wheat [53]; among other promising results for airborne LiDAR across a range of applications. Early evidence of the potential in PRG, based on a single experiment in autumn, has been presented with R^2^ = 0.79 and 0.76 for FW and DW, respectively [54].

Our hypothesis is that stand alone LiDAR is a suitable technology for measurement of vegetative biomass in PRG. This study aimed to develop a LiDAR sensor-based system, and evaluate its potential in a range of PRG phenotyping experiments targeting FW, DW and growth rate as traits related to vegetative biomass and DMY accumulation over time. Validation included data collection in the field, real-time data processing, and correlation with traits of interest.

## Methods

For this sensor platform development and validation project we assembled a multidisciplinary team combining mechanical and software engineers and field technicians and PRG breeders to develop a system targeting FW, DW and growth rate as traits associated with DMY in PRG. The team first examined spatial features, soil surface variation, and practical barriers for sensor-based field screening of PRG single-row and paired-row plots. We developed a prototype device for field-based, high throughput estimation of DMY-related traits, and automated data processing and analysis to continuously estimate DMY in real-time [54]. The system measures distances, gaps and profiles as well as introducing accuracy and precision to estimating a volumetric model of vegetative biomass in PRG. These data are difficult and costly to acquire manually or with hyper-spectral imaging techniques.

### LiDAR scanning hardware development

The original design of the in-field scanning hardware focused on adapting the LMS400 Pro (SICK Vertriebs-GmbH, Germany) LiDAR unit to enable evaluation in the field under varying plant size, ambient sunlight, moisture and wind conditions. Despite potential limitations in outdoor conditions, this LiDAR unit was selected because it provided a short operation range, scanning rate of up to 300 Hz, and relatively low beam divergence. The unit provides a typical point density of 700,000 points per square meter in the configurations of this study.

**Fig. 1:**
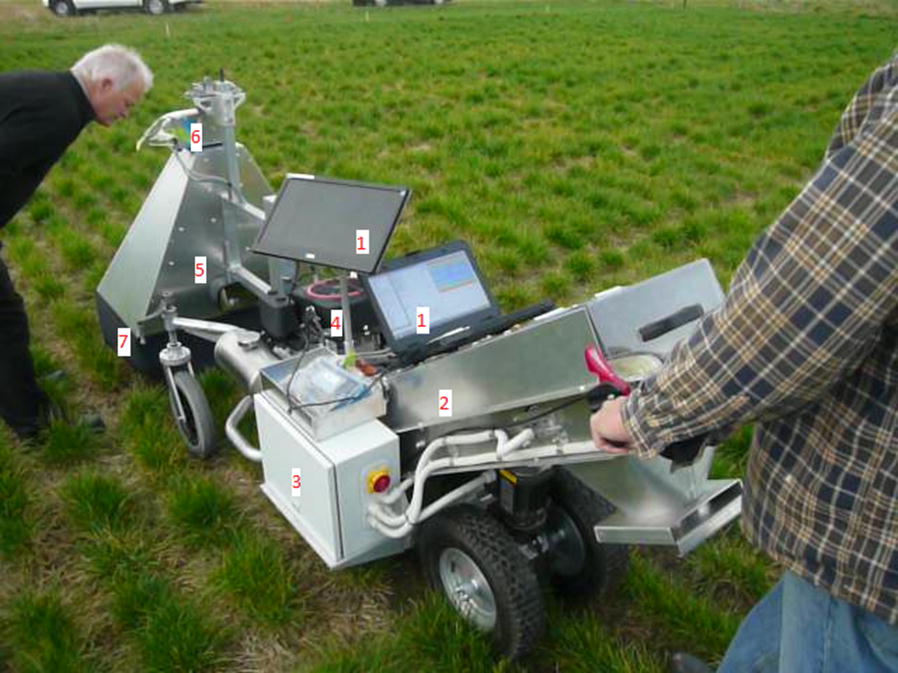
The M5 Multi-Purpose Harvesting Imaging Vehicle developed and used to collect LiDAR data as described in this publication. 1: PC screens, 2: harvester, 3: control panel, 4: generator, 5: hood, 6: LIDAR scanner, 7: blackout curtain

The LMS400 Pro is negatively affected by intense or variable light conditions. To mitigate this issue, most of the ambient outdoor light was blocked. A metal hood that reaches to within 15 cm of ground level was designed to isolate the LiDAR scanner. A black fabric curtain was added to extend to ground level and further block external light while allowing the unit to move over ryegrass plants and/or uneven field terrain (Fig. 1). The curtain was weighted to prevent it entering the canopy scan area during windy conditions. A video file of this prototype unit (named the Multi-Purpose Harvesting Imaging Vehicle or M5) in the field while scanning ryegrass plots is available at this file repository on Open Science Forum: https://osf.io/sezad/?view_only=84262a4241d048d3aad53e107677d718.

The LiDAR was powered using a 12 V sealed lead-acid automotive battery and connected to a laptop computer. Custom software was developed to capture raw data from the LiDAR unit. These data were processed in real-time and displayed on screen to the user as a dynamic point cloud during scanning. This data visualisation allowed the user to monitor data acquisition in real time.

### Data collection and processing

**Fig. 2:**
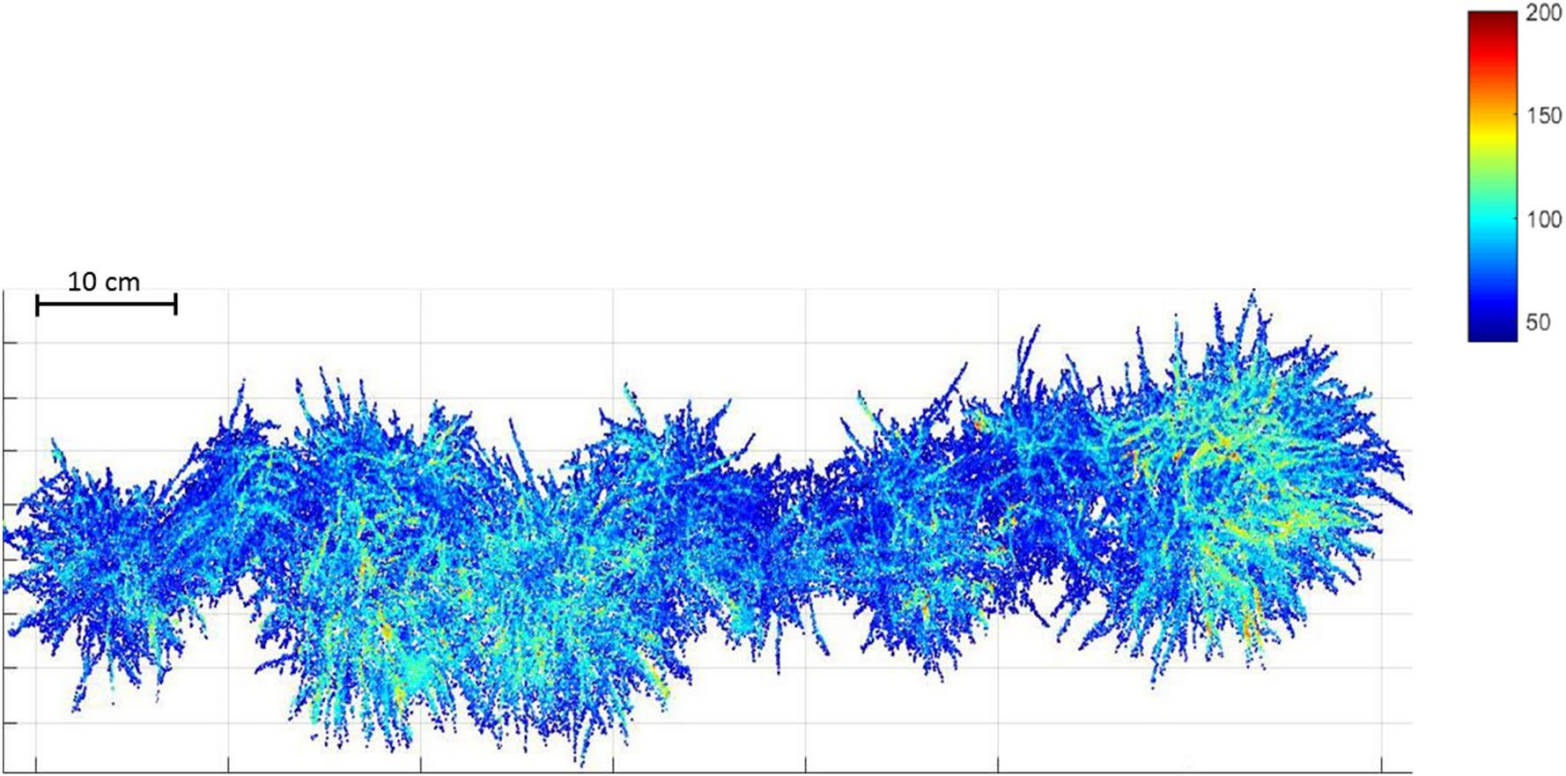
High-resolution LiDAR data of a single row of perennial ryegrass as imaged in the executable displayed in Matlab. Red color indicates the tallest grass and dark blue indicates the shortest grass as measured from the ground level in mm

Algorithms were developed to automatically segment the scanned LiDAR datastream from a sequence of raw distance measures into volumetric estimates for each single row plot of fixed dimension in a particular scanning phase (Fig. 2). For the current prototype setup, the system is capable of sequentially scanning and analyzing multiple single row plots of ryegrass. The segmentation and biomass estimation are described as follows: The LiDAR data is first pre-processed to filter noise using a moving average in the direction of the scan, along with invalid data (no signal return). The ground height is calculated for each single row plot independently, as the ground height can vary substantially between scans due to variation in ground level in field trials. The ryegrass component of the data is then segmented from the surrounding soil background by height, with the operator supplying a standard virtual cutting height above ground to mimic grass segmentation resulting from mechanical defoliation. The LiDAR data in each ryegrass region was then further processed to derive the biomass estimate for each plot. This was done by converting the 2D LiDAR height measures into a 3D surface, then integrating the surface over the grass region at the virtual cutting height which was standardized across the trial.

The final software and data processing configuration allows the user to manually correct instances of sparsely populated and also poorly separated plots. These situations can arise as artifacts of planting errors, severe weed incursion, plant death, or lax management of plant growth and defoliation cycles over the course of a multi-year field trial. The user is able to capture, monitor, and process LiDAR data in the field and acquire real-time, non-destructive biomass estimates, which we term LiDAR Volume (LV) at a speed of 0.5 m/s. The LV value is a unitless volumetric index whose calculation has been optimized for computational efficiency and end-user preference over the course of these experiments, and is calculated as the integral of the LiDAR scans, multiplied by the distance travelled and then divided by the number of scans:

$$ LV = \left[ {\sum \left( {\int LIDAR\; Scans} \right) \times distance} \right] \div Number\;of\;scans $$

## Field experiments

Four independent field experiments were conducted, with entries and locations as summarized in Table 1.

**Table 1:** Summary of the four field experiments conducted to develop and evaluate a LiDAR-based approach to estimating foliage yield traits in perennial ryegrass

Exp.	Location	Entries	Design	# Plots	Traits	Sown	Measured	Material
1	− 43.45, 172.19	120	58C × 30R	360	FW, DMY, LV	May 2016	Sept. 2017	HS families
2	− 43.62, 172.46	12	44C × 6R	36	Growth rate, LV	March 2015	Autumn 2016	Cultivars
3	− 43.63, 172.47	32	47C × 33R	96	Growth rate, LV	May 2013	Spring 2016	HS families
4	37.77, 175.31	190	18C × 35R	630	FW, LV	Spring 2017	2018–2019	BL

*FW* fresh weight, *DMY* dry matter yield, *LV* LiDAR Volume, *BL* breeding lines

### Experiment 1: foliage yield estimation in spring

The goal of this experiment was to initially evaluate the LiDAR platform’s ability to estimate vegetative FW and DW as expressed in single row plots of PRG after a single regrowth phase following defoliation.

LiDAR scans were completed on 26 September 2017 on a subset of 360 plots in a large, ongoing trial consisting of 1740 single row diploid PRG plots of half-sibling (HS) families. The experimental design was a row-column (58 columns × 30 rows) within which each HS family was replicated three times, and which included repeated checks. The 360 scanned plots were three replications of 120 HS families, comprised of all 30 plots within each of 12 columns evenly spaced across the trial of 58 columns, in order to capture a broad sample of spatial and phenotypic variation within the trial. Each plot was 1 m long and sown on 30 cm centres in May 2016 at Agriseeds Research Station (− 43.45, 172.19) near Darfield, New Zealand. The trial had been mechanically defoliated 12 times prior to this experiment. The most recent defoliation prior to the experiment was 20 August 2017. Prior to scanning the trial was weeded to ensure only PRG was present. Both FW and DW data were collected from harvested samples, with DW samples dried at 80 °C for at least 48 h prior to data collection. The LVs were analysed and compared to FW and DW by correlation.

### Experiment 2: recurrent scanning for growth rate at lower temporal resolution

Experiment 2 was conducted in a field trial of 86 single row plots of 28 ryegrass cultivars each replicated three times except one with five replications. Each row was 2 m long and planted by seed on 50 cm centres in March 2015 at Kimihia Research Centre (− 43.62, 172.46) near Lincoln, New Zealand. The trial had been defoliated by grazing eight times prior to this experiment. The most recent defoliation prior to the experiment was 23 March 2016.

The LiDAR platform described above was used to scan 36 plots of the 12 target cultivars in autumn 2016 at 41 days, 55 days and 83 days after the last defoliation to test the ability of the system to detect growth rate at a low temporal resolution. These 12 cultivars were selected to represent a broad set of ryegrass cultivars with a diverse moisture contents, ploidy level, flowering time, and habit with minimal redundancy. Immediately after scanning at day 83, each plot was mechanically harvested to a residual height of 3 cm above ground level. The FW and DW data were collected from harvested samples of 36 single row plots consisting of 3 replicates of 12 cultivars by weighing at harvest time for FW, and oven drying and recording DW of the sample for each plot. The LVs were analysed and compared to the FW yield data to assess correlation. Using LV obtained from calibration against field harvests on day 83, growth estimates were made for days 41, 55 and 83; and growth rate was estimated for each cultivar.

### Experiment 3: recurrent scanning for growth rates at high temporal resolution

The purpose of this experiment was to evaluate the ability of our LiDAR system to recurrently scan and estimate growth in the same single row plots over time periods as short as 48 h within a typical spring regrowth phase for PRG. Mechanical defoliation was completed on 19 October 2016 (southern hemisphere spring) for 96 of 1551 PRG single-row plots sown in May 2013 (the Lincoln STD treatment in Faville, Ganesh [55]) and growing in a field trial similar to the experiment above. These plots were three replicates of 32 genotypes scanned starting October 19, 2016 every 2 to 5 days over a total period of 26 days during the reproductive spring growth phase, while growing under non-irrigated conditions at the AgResearch Farm at Lincoln, New Zealand (− 43.63, 172.47). A total of nine scans were made during this period at 0, 2, 6, 9, 14, 16, 19, 21 and 26 days regrowth to acquire 864 plot × scan combinations. The LV data were used to estimate growth rate as outlined below. There was no mechanical harvest during this experiment, so FW and DW data were not collected.

### Experiment 4: recurrent scanning for seasonal growth estimation in paired-row breeding trials

The purpose of this experiment was to evaluate the LiDAR system in a new region of New Zealandusing a modified field trial design with paired-row plots (Additional file 1: Fig. S2). In spring 2017, 190 PRG breeding lines were sown in a breeding trial at Ruakura, New Zealand (− 37.77, 175.31). A row–column experimental design with three replicates was used. Each replicate also contained 20 repeated checks. Each plot was a paired-row and consisted of two adjacent, 2-m-long rows sown with a cone seeder at 20 kg/ha. The space within double rows of each plot were spaced 15 cm apart, and spacings between outside rows of adjacent plots were 40 cm. The ends of plots within a column were spaced 30 cm apart. Each column consisted of 18 plots, and there were 35 rows, comprising the 630 plots in total for the field trial.

The trial was managed as a monoculture and rotationally grazed by a flock of 100–150 sheep when 2400–3000 kg DM/ha were visually estimated to have accumulated. Herbicide was applied when required to control broad leaf weeds and C4 grass invasion. Nitrogen fertiliser was applied using Urea (46–0–0–0) at 200 kg N/ha/year in small increments following grazing.

Pre-grazing, all plots were scanned using LiDAR between March 2018 and January 2019. After the LiDAR scan, 20% of plots (126 plots) were sampled by mechanical defoliation using a rotary lawn mower, weighed and their FW recorded. The location of the 126 plots varied at each harvest so no single plot was mechanically harvested more than once in five consecutive harvests—this methodology was used to expose breeding lines to as much ruminant grazing pressure as possible and help mitigate bias in vegetative persistence between breeding lines. Post-grazing, nurseries were mown to a height of 4 cm to homogenize the pasture cover and all cut herbage was removed from the trial sites.

At scanning, the LiDAR unit travelled down each column and back up the adjacent column in a serpentine pattern for the entire trial. Data was saved as Text file at the end of each column and processed in a similar manner. The processing software parameters were set to; Scan length—36 m, Segments per scan—18, Row spacing—550 mm, and cutting height—40 mm. Manual plot alignment was required from time-to-time to distinguish the start and end of plots.

Correlations were observed for only the plots where FW data were collected at each harvest. Mean FW per plot varied from harvest to harvest.

### LV and yield data analysis

Initial analysis of FW and DW and LV data was carried out using Microsoft Excel to assemble and summarise the data. Correlations of FW and DW data with LVs were calculated using GraphPad Prism version 8.00 (GraphPad Software, La Jolla, California, USA). Percent dry matter (%DM) was calculated as (DMY/FW) × 100. For Experiment 1, coefficient of variation ($CV = \sigma /\mu$) were estimated for FW, DW, and scanning measurements among cultivars where σ is standard deviation and µ is sample mean. Also, normalized covariance matrix (− 1 to 1) was calculated to observe any cultivar effect on the slope and intercept of this comparison.

Relative growth rate over the period of measurements was calculated as $RGR = \left( {\ln Y2 {-} \ln Y1} \right)/\left( {t2 - t1} \right)$ [where RGR is relative growth rate, Y2 is yield at time point 2 and Y1 is yield at time point 1 and t2 − t1 is the time interval (days)] [56] was also calculated using LVs as yield for both experiments 2 and 3.

## Results

A series of experiments validated a ground-based mobile scanning LiDAR platform, and the potential for high accuracy and precision measurement of FW and DW in single-row and paired-row plots of PRG in field trials. An initial assessment of the system’s ability to measure growth rate in PRG was also very informative as presented below.

### Experiment 1: foliage yield estimation

**Fig. 3:**
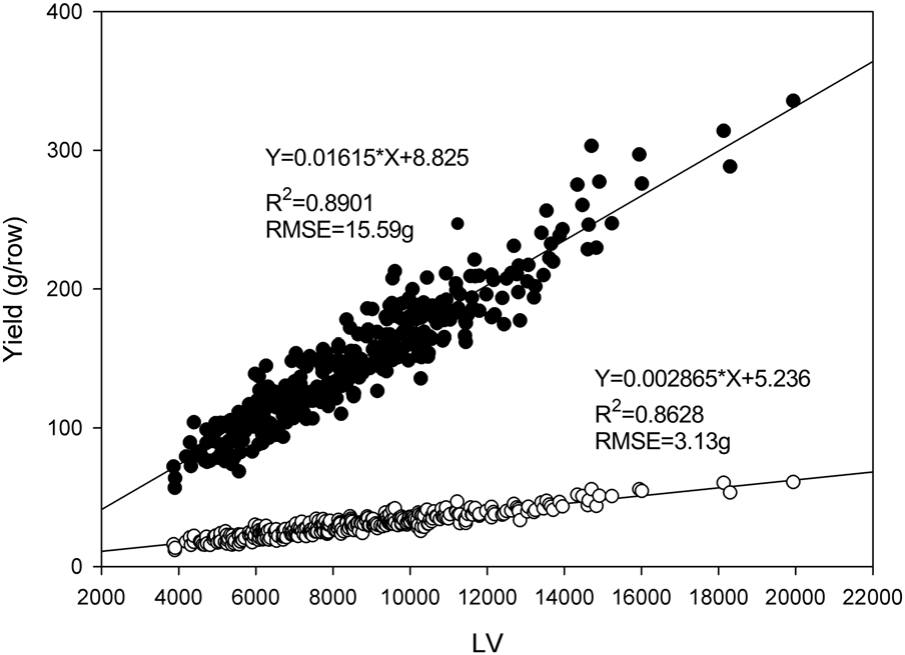
LiDAR Volume (LV) plotted against harvested dry weight (DW, open symbols) and fresh weight (FW, closed symbols) yields of 360 rows of a diploid perennial ryegrass field experiment measured in spring regrowth near Darfield, New Zealand. Correlation of LV with these data are R^2^ = 0.89 and 0.86 for FW and DW, respectively

In the field trial plots measured in spring, FW and DW estimates ranged from 57 to 335 g/row and 12 to 61 g/row, respectively. The LVs ranged from 3.86 × 10^3^ to 19.94 × 10^4^. Correlation of LV with FW and DW were R^2^ = 0.89 and R^2^ = 0.87, respectively (Fig. 3).

### Experiments 2 and 3: recurrent scanning for growth rate estimation

Recurrent scanning over three dates in three replicates of 12 cultivars in a field trial were acquired and summarized by entry. Correlations between FW and LV for each cultivar is listed in Table 2. After normalized covariance matrix analysis, all slopes between cultivars and the regression parameters resulted in the same value close to − 1 (− 0.9926). The coefficient of variation data showed the similarity in the spread of the LV with DW data more than with FW and  %DM (Table 3). No pattern for difference in FW, DW, %DM or LV between tetraploid and diploid cultivars was observed (Table 3). The data reveal accumulation of LV over time, and inspection of these plots suggest there may be differences in growth rate and pattern among cultivars (Additional file 1: Fig. S1).

**Table 2:** Comparison and regression analysis of fresh weight yield and LiDAR Volumetric Estimate in 12 cultivars of perennial ryegrass

CV	Slope	Y-intercept	X-intercept	R^2^	Slope deviates from zero (*P*)
1	251,687 ± 18,797	− 24,786,555 ± 2,701,665	98.48	0.9945	0.0475*
2	367,976 ± 9248	− 36,712,222 ± 1,329,168	99.77	0.9994	0.016*
3	268,352 ± 31,270	− 27,599,719 ± 4,494,494	102.8	0.9866	0.0739
4	296,257 ± 50,781	− 29,848,658 ± 7,298,879	100.8	0.9715	0.1081
5	375,228 ± 14,687	− 39,844,763 ± 2,111,043	106.2	0.9985	0.0249*
6	254,370 ± 8264	− 25,279,526 ± 1,187,740	99.38	0.9989	0.0207*
7	213,756 ± 2424	− 21,271,669 ± 348,411	99.51	0.9999	0.0072**
8	287,494 ± 957.5	− 28,144,506 ± 137,617	97.9	1	0.0021**
9	288,594 ± 2449	− 26,977,390 ± 351,961	93.48	0.9999	0.0054**
10	318,106 ± 5578	− 33,444,196 ± 801,692	105.1	0.9997	0.0112*
11	333,438 ± 16,568	− 34,701,094 ± 2,381,281	104.1	0.9975	0.0316*
12	212,148 ± 7899	− 19,647,030 ± 1,135,327	92.61	0.9986	0.0237*

Values for slope and Y-intercept represent mean ± SD

* Significant; ** highly significant

**Table 3:** Mean coefficient of variation for four measured traits in 12 cultivars of perennial ryegrass

Cultivar	FW (g/row)	DW (g/row)	%DM	LV	Ploidy
1	525.00	154.15	0.29	16,841,584	2x
2	655.00	190.14	0.29	24,446,478	4x
3	391.42	130.49	0.33	17,199,440	4x
4	579.75	188.97	0.33	18,919,565	2x
5	498.39	141.70	0.28	22,324,398	2x
6	403.11	131.24	0.33	17,012,703	4x
7	370.61	126.08	0.34	14,231,352	2x
8	499.67	147.76	0.30	19,571,709	2x
9	514.49	171.25	0.33	20,948,934	2x
10	522.54	158.45	0.30	19,316,282	4x
11	475.96	161.89	0.34	20,783,519	2x
12	317.75	108.53	0.34	15,633,334	2x
CV	19.72%	16.59%	7.13%	15.43%	–
σ	94.57	25.04	0.03	2,923,314.65	–
µ	479.47	150.88	0.31	18,935,774.83	–

**Fig. 4:**
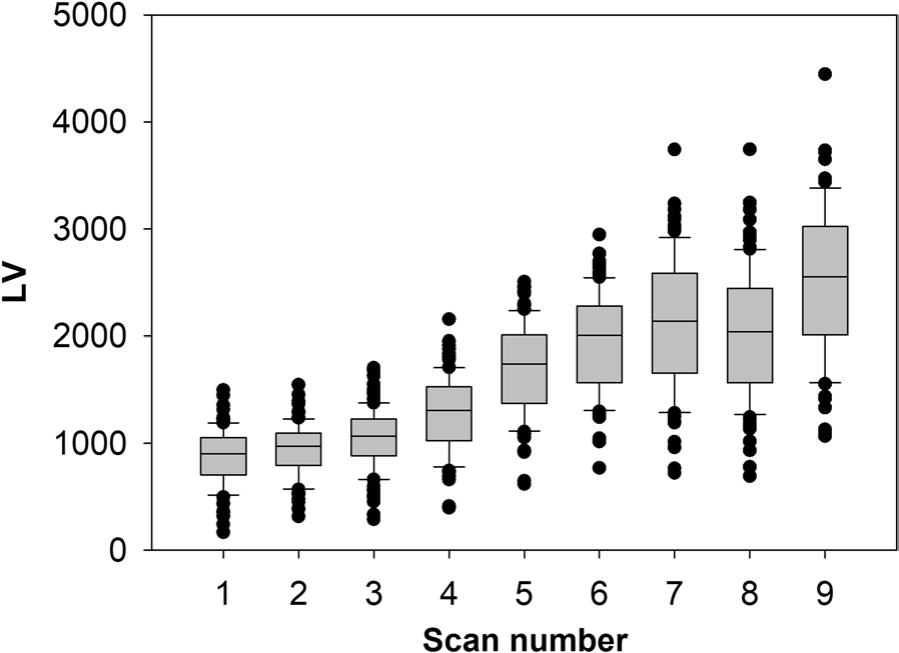
Accumulation of LiDAR Volumet (LV) in single-row plots of perennial ryegrass in Lincoln, New Zealand recurrently measured at 0, 2, 6, 9, 14, 16, 19, 21 and 26 days (corresponding to scans 1–9 on X axis) of a regrowth phase in spring

To explore the potential for LV to measure growth rate at higher resolution, an experiment was conducted with LV data collected in 96 plots every 2 to 5 days over a span of 26 days (Fig. 4). The data generally reveal a pattern of increasing LV over time, with some variation due to either changes in field conditions or technical factors influencing LV. While showing an increase in LV of up to 4% per day and general trend of increasing growth, the measurements showed a period of contraction in LV, followed by a marked increase at the final measurement (Fig. 4). While this may be attributed to variation in temperature or rainfall patterns, we cannot draw a firm conclusion as to the causal factors behind the contraction in LV at day 21.

### Experiment 4: recurrent scanning of paired-row plots across seasons

**Fig. 5:**
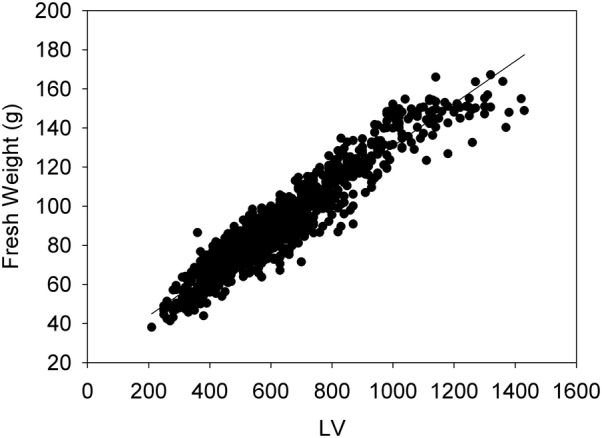
Correlation (R^2^ = 0.90) of LiDAR Volume and Fresh Weight data across 1008 observations in a paired-row perennial ryegrass field experiment in Ruakura, New Zealand, for recurrent harvests between April 2018 and January 2019

Across eight harvests of vegetative regrowth 126 paired-row plots in autumn, winter, spring and summer seasons in 2018–2019, correlations between LV and FW ranged from R^2^ = 0.67 to 0.93 (Table 4). In aggregate, the correlation of all 1008 LV and FW observations is R^2^ = 0.90 (Fig. 5). Given the prior results, there are two instances of unexpectedly low correlation. The first, in July 2018 is attributable to use of the LiDAR unit without the light exclusion curtain. The second, in December 2018, was at a time of particularly high growth, indicating there may be upper limits to the amount of grass biomass that can be accurately measured in the current configuration. However, the amount of vegetative growth measured in the December 2018 dataset are above the maximum recommended for pasture management under rotational grazing.

**Table 4:** Correlation between LiDAR scan data and harvested vegetative biomass of paired-row ryegrass plots in a breeding trial in Ruakura, New Zealand across nine monthly measurements with an annual cycle

Harvest	R^2^	LV	FW (g)
Mar-18	0.80	105 ± 14.3	750 ± 130
Jul-18	0.68	82 ± 10.0	640 ± 90
Aug-18	0.81	84 ± 11.4	520 ± 110
Sep-18	0.81	98 ± 15.5	680 ± 110
Oct-18	0.93	75 ± 17.6	510 ± 113
Nov-18	0.81	68 ± 13.6	450 ± 90
Dec-18	0.67	137 ± 14.7	1030 ± 170
Jan-19	0.81	73 ± 13.9	440 ± 100

LV and FW data are mean ± standard deviation

*LV* LiDAR Volume, *FW* fresh weight

## Discussion

Vegetative biomass in crops and forages can be measured by measuring the FW and DW [35]. However, this will be a destructive method thus limiting measurements to the end of growth period only and are generally expensive and labour-intensive. Similarly plant growth rate is usually estimated by destructive measurements at the end of growth period [57] or computer modelling [58]. This suggests a potentially crucial role for the LiDAR scanner and software as key components to improve the quality, efficiency and resolution of future yield and growth rate measurements in the field. This is particularly the case in perennial pasture plants where DMY accumulation over many cycles of regrowth and defoliation is an economic trait of substantial interest.

While, in the past, LiDAR tools have been suggested to have the measurement capability of different forage species in combination with other sensors [59], there are limitations for each tool and as an example, devices, systems and methods provided in patent US20160084635A1 [47, 60] need to acquire and weight data across a range of sensors to be able to estimate DMY. They also take spot or small area readings and therefore cannot be used for rows or spaced plants.

Results obtained from manual measurements in this study suggest that a broad range of FW and DW levels can be directly measured by LiDAR in PRG. However, there may be upper limits after which the accuracy of the current algorithm decays.

Both FW and DW in rows are correlated to DMY accumulation over time in complex swards under a wide range of management and defoliation regimes. In experiment 1, we observed a higher correlation coefficient between FW and LV than between DW and LV. This is expected given the LV is a volumetric measure which will be a function of both DM and water content in the scanned biomass. The potential for LV to estimate %DM, even at a lower precision, would overcome a significant bottleneck in plant breeding, and warrants further investigation.

The equal value of normalized covariance matrix for all 12 cultivars indicates high dependence among parameters. This was an expected result given the strong relationship of LiDAR, FW and DW. Greater accuracy of the system to scan biomass alone will affect these parameters in the estimation of FW by regression if that is the chosen application in the future.

The ability to generally attain correlations greater than R^2^ = 0.80 across sites, seasons, levels of regrowth and age of trials offers initial evidence of the system’s reliability and utility. Even when used in a non-design configuration without a light exclusion and on double rows, the correlation was R^2^ = 0.68 which compares favorably with the precision of other sensor systems and visual observations.

However, the need to further explore the relationship between LV and changes in environmental conditions, and the relationship between LV and vegetative biomass at the upper and lower bounds of growth remain an area for ongoing research. Based on results obtained in our experiments, we suggest that LiDAR is a consistent, repeatable measurement tool and produces valuable data for measuring DMY in PRG.

The advantage of information obtained from coefficient of variation calculations is that despite the large standard deviation in LV compared with the other three datasets (FW, DW and %DM), the coefficient of variation data compares variability across different variables with the same relative scale. It is, therefore, an indicator of the same scale of data dispersion for LV and dry matter measurements. The study group’s growth rate was not obviously affected by the ploidy level, endophyte type, or the hybrid nature of cultivars. This confirms the potential of LV for directly measuring DMY via FW and DW, and the generic applicability of information obtained from this type of data across different types of ryegrass. However, further research into the effect of environment, management and genotype may reveal opportunities to specifically improve the LV algorithm for specific cases.

The results obtained from Experiments 1 and 4 compare favourablly with the results obtained from studies in crop species such as wheat and maize, which show R^2^ range of 0.72–0.84 between vegetative biomass dry matter and LV [52, 61, 62]. These results also compare favourablly to other reports in pasture species where multi-sensor systems were used typically resulting in correlations of R^2^ less than 0.80 [48, 60]. Despite growth rate being a trait of inherent interest, to date there have been no tools available for breeders to precisely quantify it. The data presented here indicate the LiDAR system may provide a means for this trait to be measured at a temporal resolution meaningful for plant breeding and agronomy applications. A limitation of the data in Experiments 2 and 3 are that we were unable to collect concurrent FW or DW data due to limitations of plant material available. Also, the volumetric fluctuation observed at very short time frames (days) in relation to environmental variation and inherent technical variation require further exploration before drawing conclusions as to any causal factor(s) affecting the results.

To further explore the potential for assessment of growth rate at high temporal resolution, it would be valuable to screen highly replicated trials with divergent populations of ryegrass over multiple fixed time points, while simultaneously conducting visual assessment and destructive harvest for FW and DW measurement on a small number of replicates at the end of each growth period. Further investigating the LiDAR’s capability to measure % water content would also be an interesting prospect although we cannot be certain of causes of contraction in LV during a regrowth phase. An experiment to measure FW and DW at well-watered and drought stressed sites to investigate the possibility of measuring stress via wilting and reduction in LV would create opportunities for more efficient selection of populations with adaptive response to abiotic stress. The first suggestion will provide data to explore the potential of LiDAR for increasing accuracy. The last suggestion would provide a means for potentially developing an inexpensive, reliable and high throughput tool for stress tolerance/susceptibility estimation before chlorosis, which is simply not achievable using visual spectrum cameras only.

Data obtained in this study are based on ryegrass grown in single and paired-rows, and not in agronomic plots or mixed swards. There is evidence that the yield of ryegrass may change in response to the accompanying plant species [58], therefore the potential for LV scanning also needs to be assessed in small monoculture and mixed-species plots and in mixed swards, using white clover and other species in field experiments. However, to observe the complete picture of DMY accumulation in ryegrass, the LiDAR must be ultimately used over a range of years, sites, and grazing management regimes to get a full picture of DMY accumulation and plant vegetative persistence over time, as it is a perennial species with a longer breeding and utilization cycle [58].

The development of LV scanning for vegetative biomass and incorporating factors that may affect DMYin the field into the algorithm is a starting point for further investigation that could be well extended to other grass species across a range of breeding and agronomic applications. It also provides an opportunity to more efficiently collect data of value in genomic selection and other advanced breeding strategies, and to facilitate discovery of genetic loci influencing this trait.

Dry matter yield can be divided into a few simpler traits or sub-traits. No significant FW, DW or LV difference between tetraploids and diploids in these experiments was contradictory to differences in cell size and suggestions otherwise by [58]. Number of tillers, another candidate sub-trait of interest and which directly correlates with total forage yield [63], may be possible to measure by counting if needed by modification of the current algorithm developed in this study, though it was not tested in this study and remains speculative. Both sub-traits are considered a priority for more detailed studies in the future.

Further, measurements in our experiments indicate that we can use LV to estimate FW and DW with high accuracy within the target range of vegetative biomass suited to grazing of pasture, but may need refinement for forage conservation. This provides a means to address the suggestion [64] that the current tools need to be complemented by a tool that can directly measure DMY. However, measuring vegetative biomass and morphology in spaced ryegrass plants still needs further software and probably hardware improvements such as Real Time Kinematic Global Positioning System, although they share the same principles with yield measurement in rows such as the presence of bare soil between plants and so are likely amenable to LiDAR-based approaches.

## Conclusions

A LiDAR-based system for real-time non-destructive measurement of vegetative biomass in relation to DMY accumulation in PRG single and paired-row plots was validated under different scenarios. We demonstrated the potential and identified some limitations of a single LiDAR sensor for generalized estimation of FW, DW and growth rate in field experiments across a range of seasons, sites and ages. We suggest that some discoveries in this study, such as the potential for the LiDAR system to detect variation in LV at a high temporal resolution may improve breeding processes in the future by enabling measurement of growth rate. Although our results support phenomics research findings reported in crops and other forage grasses, this is the first time a LiDAR-based tool has been shown to quantify DMY or biomass data for PRG field experiments in real-time and at high accuracy, generally R^2^ > 0.8. The suggestions provided for future studies including a closer examination of the effects of environment, management and genotype on accuracy may further improve the quality and resolution of LiDAR data for agricultural purposes, and also increase our knowledge of this sensor/tool and will lead to high throughput and more effective breeding programs, agronomy and farm management technologies.

## Additional files

**Additional file 1: Fig. S2.** Field image of perennial ryegrass experiment in paired-row plot configuration in Ruakura, New Zealand, used for Experiment 4 data collection.

**Additional file 2: Fig. S1.** Fresh weight estimates and growth rates in 12 cultivars of perennial ryegrass in a replicated field experiment, based on LiDAR scans at three timepoints measured in a spring regrowth phase.

## Data Availability

The datasets used and/or analysed during the current study are available from the corresponding author on reasonable request.

## Abbreviations

%DM: percent dry matter
DMY: dry matter yield
DW: dry weight
FW: fresh weight
HS: half-sibling
LiDAR: light detection and ranging
PRG: perennial ryegrass
