# Supplementary figures and images for: Real-time, non-destructive and in-field foliage yield and growth rate measurement in perennial ryegrass (Lolium perenne L.)
Source: Plant Methods. 2019 Jul 10;15:72. doi: 10.1186/s13007-019-0456-2 (PMC6617592; doi:10.1186/s13007-019-0456-2)

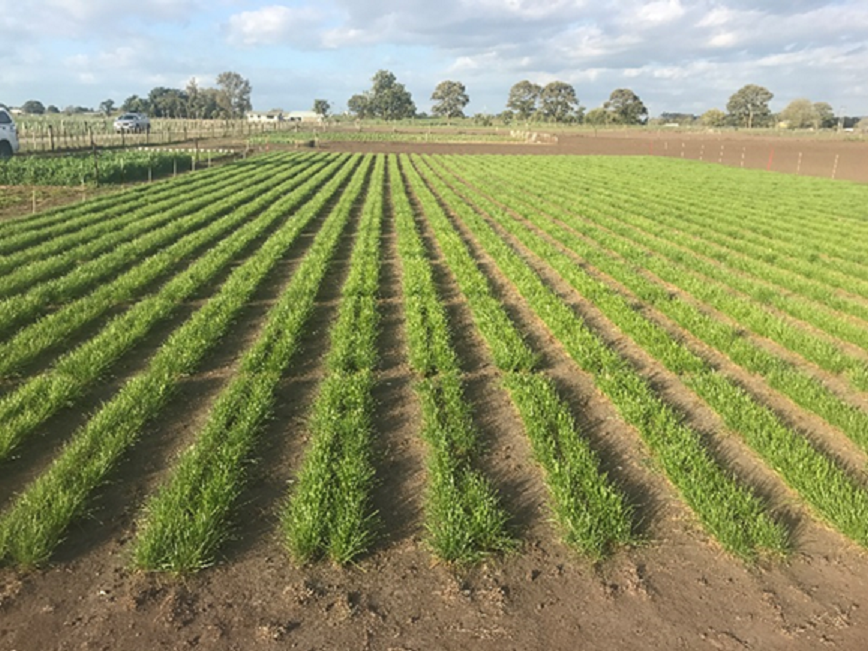

Supplement: Supplementary file 1 — Additional file 1: Fig. S2. Field image of perennial ryegrass experiment in paired-row plot configuration in Ruakura, New Zealand, used for Experiment 4 data collection. [file 13007_2019_456_MOESM1_ESM.tif]

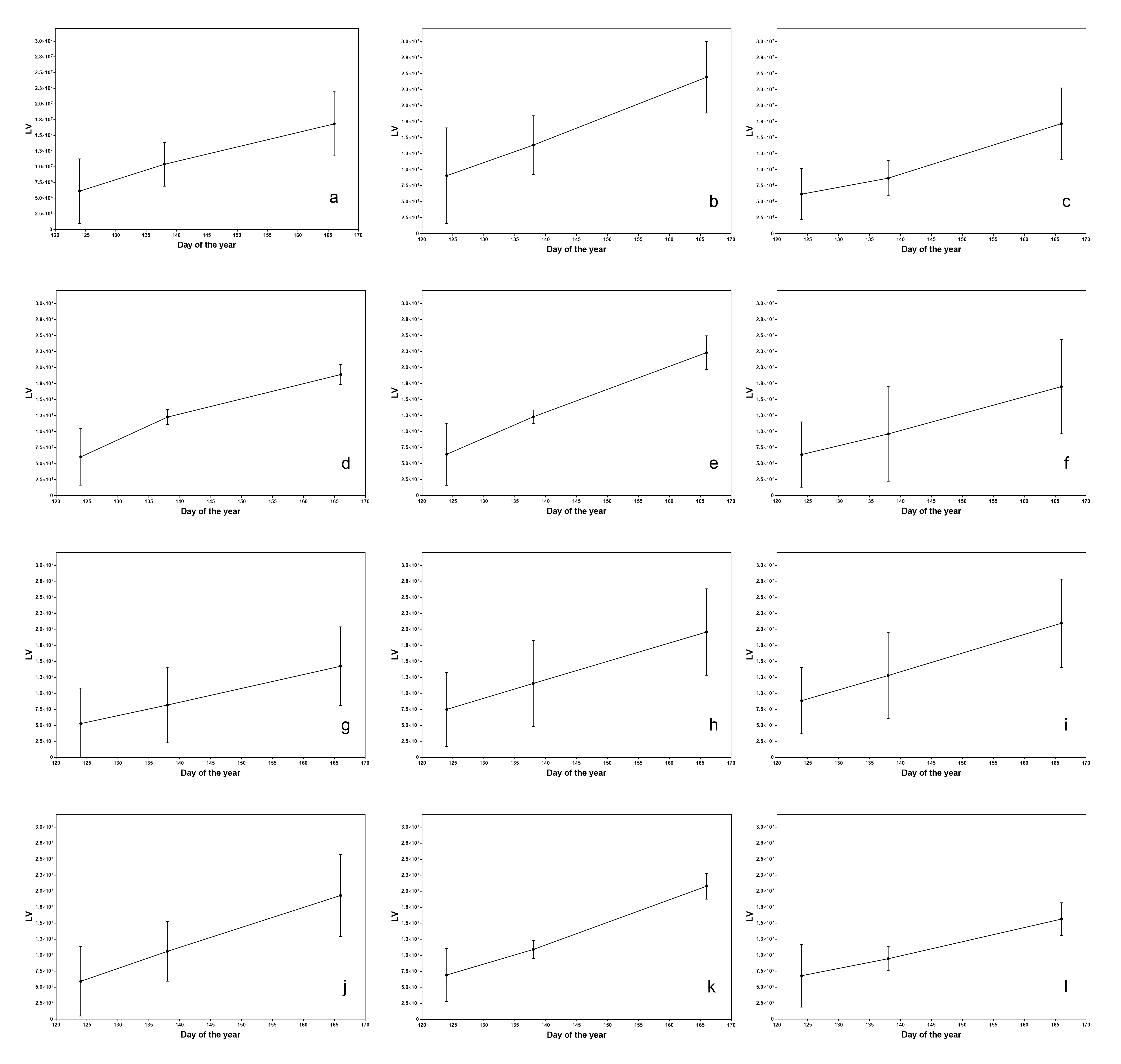

Supplement: Supplementary file 2 — Additional file 2: Fig. S1. Fresh weight estimates and growth rates in 12 cultivars of perennial ryegrass in a replicated field experiment, based on LiDAR scans at three timepoints measured in a spring regrowth phase. [file 13007_2019_456_MOESM2_ESM.tif]
